# Wnt Signalling‐Activated EN2 Promotes the Progression of Glioblastoma by Upregulating Fatty Acid Synthesis Metabolism

**DOI:** 10.1111/jcmm.70726

**Published:** 2025-09-01

**Authors:** Junjun Zhang, Shengjun Zhou, Zifeng Dai, Fanyong Gong, Jianfei Zhang

**Affiliations:** ^1^ Department of Neurosurgery The First Affiliated Hospital of Ningbo University Ningbo Zhejiang People's Republic of China

**Keywords:** EN2, fatty acid synthesis metabolism, glioblastoma, SREBP1, TCF4, Wnt signalling pathway

## Abstract

Glioblastoma (GBM) is the most aggressive primary brain tumour, with limited treatment options and a propensity for rapid development of resistance to therapies. Previous studies have indicated that fatty acid metabolic reprogramming is a critical marker of tumour progression and plays a significant role in the proliferation and migration of cancer cells. However, research on fatty acid synthesis metabolism in GBM is relatively limited, and the underlying mechanisms warrant further investigation. In this study, we identified a significant correlation between the expression of Engrailed 2 (EN2) and poor prognosis in GBM patients. Both in vivo and in vitro experiments demonstrated that EN2 promotes GBM progression and facilitates fatty acid metabolic reprogramming. Mechanistically, EN2 activates the expression of Sterol Regulatory Element‐Binding Protein 1 (SREBP1), thereby enhancing the fatty acid synthesis metabolic pathway and contributing to tumour resistance. Furthermore, we found that EN2 is primarily regulated by the Wnt signalling pathway and T‐cell factor 4 (TCF4). Targeting EN2 enhances the efficacy of chemotherapy in GBM and prolongs survival in mouse models. Overall, our findings suggest that EN2 represents a potential therapeutic target for GBM and underscores its role in promoting fatty acid synthesis metabolism in GBM cells.

**Abbreviations**
EN2: engrailed 2
GBM: glioblastoma
MCT1: monocarboxylate transporter 1
SREBP: sterol regulatory element‐binding protein
TMZ: temozolomide

## 1 Introduction

Glioblastoma (GBM) is the most aggressive malignant brain tumour. The current main treatment modalities include surgery, temozolomide chemotherapy (TMZ) and radiotherapy [1, 2]. However, the five‐year survival rate for patients after treatment is less than 5%, and the prognosis is poor [3]. Metabolic reprogramming plays an important role in tumour progression [4]. Studies have shown that GBM cells require fatty acid metabolism to obtain energy, membrane components and signalling molecules, thereby promoting their own proliferation. In contrast, blocking lipid production in cancer cells can observably inhibit GBM progression [5, 6]. Therefore, fatty acid metabolism is an important pathway in the development and treatment resistance of GBM, providing new insights into the search for effective treatment strategies for this condition.

Engrailed 2 (EN2), a transcription factor with a homeodomain structure, plays a crucial role in embryonic neural development [7, 8]. Multiple studies have indicated that EN2 is highly expressed in various cancer cells and tissues and is associated with the occurrence and progression of different cancers [9, 10, 11]. For example, EN2 plays a key role in promoting colorectal cancer progression primarily through the regulation of the expression of CCL20, which in turn promotes the proliferation and migration of colorectal cancer cells [9]. Furthermore, EN2 is negatively regulated by miR‐605 in prostate cancer. Downregulating miR‐605 leads to a significant upregulation of the expression of EN2, promoting the proliferation and invasive capacity of prostate cancer cells [10]. This indicates that EN2 plays an important role in cancer occurrence and progression, but its main mechanisms of action in GBM remain unclear.

Fatty acid metabolism reprogramming is a critical hallmark of tumour progression, as fatty acids are essential for energy acquisition, membrane biosynthesis and intracellular signalling in cancer cells [12, 13]. The sterol regulatory element‐binding protein (SREBP) family comprises important transcription factors that regulate the expression of genes associated with lipid synthesis and uptake, thereby playing a significant role in fatty acid metabolism [14]. In mammals, SREBP is encoded by two genes, SREBP1 and SREBP2, with SREBP1 yielding two isoforms: SREBP‐1a and SREBP‐1c [15]. Numerous studies have demonstrated that SREBP1 is aberrantly expressed in various cancers, including pancreatic, prostate and breast cancer, where it assumes critical functions [16, 17, 18]. For example, high glucose levels have been shown to upregulate SREBP1 expression while inhibiting apoptosis and autophagy, thus promoting the proliferation of pancreatic cancer cells [16]. Moreover, berberine can inhibit lipidogenesis mediated by the SCAP/SREBP‐1 signalling pathway, leading to the inactivation of the Wnt/β‐catenin pathway, which in turn suppresses the proliferation and growth of colon cancer cells [19]. Despite the well‐established roles of SREBP1 in other cancer types, research on fatty acid metabolism reprogramming in GBM remains relatively scarce, indicating a pressing need for further investigation.

The Wnt/β‐catenin pathway is a highly conserved signalling pathway involved in embryonic development and plays important roles in various physiological processes [12]. Additionally, the Wnt pathway is involved in the progression of several diseases, including cancer [20]. However, abnormalities in the Wnt/β‐catenin pathway due to epigenetic modifications or other factors often lead to the pathway and its downstream target genes being aberrantly activated [12]. Furthermore, an increasing number of studies have shown that dysregulation of Wnt signalling is associated with the progression of various cancers, including hepatocellular carcinoma, pancreatic ductal adenocarcinoma and breast cancer [14, 15, 16]. For example, autophagy can promote the expression of monocarboxylate transporter 1 (MCT1) by activating Wnt/β‐catenin signalling, further promoting the metastasis and glycolysis of HCC cells [14]. Notably, there is a close relationship between the Wnt/β‐catenin pathway and SREBP1, both of which play significant roles in cancer progression. SREBP1 enhances the proliferation of human oesophageal squamous cell carcinoma (ESCC) cells and epithelial‐mesenchymal transition by activating the Wnt/β‐catenin signalling pathway induced by SCD1 [21]. Furthermore, in colorectal cancer cells, the interaction between SREBP1 and Wnt/β‐catenin plays a central role in promoting cancer cell proliferation and metastasis [22]. However, the expression and functional roles of Wnt/β‐catenin signalling in GBM remain unclear.

In this study, we demonstrate that EN2 can promote GBM progression in vitro and in vivo, thereby activating fatty acid metabolism in cancer cells. Furthermore, EN2 is primarily positively regulated by the upstream Wnt signalling pathway. It upregulates the expression of SREBP1 to activate fatty acid metabolism in GBM cells, influencing metabolic reprogramming and subsequently promoting cancer cell proliferation. Overall, our research suggests that EN2 is a promising therapeutic target for overcoming treatment resistance in GBM.

## 2 Materials and Methods

### 2.1 Tissue Samples

A total of 33 GBM tissue samples and their adjacent non‐cancerous tissue samples were collected from patients at the First Affiliated Hospital of Ningbo University. This study complies with all relevant ethical regulations regarding research involving human participants and has been approved by the First Affiliated Hospital of Ningbo University Institutional Review Board (Approval No. 2023127A). We adhered strictly to ethical guidelines during the sample collection process, ensuring that all participants provided informed consent to protect their privacy and rights.

### 2.2 Cell Culture and Treatment

HEB, A172, LN18, U87, U118 and other cell lines are derived from the American Type Culture Collection. All cells were cultured in DMEM medium supplemented with 10% FBS, 1% penicillin–streptomycin and 1% glutamine. They were maintained in a 5% CO_2_ incubator at 37°C. Cultured cells were grown to 75%–85% confluence and then treated with various concentrations of cinobufotalin, NDI‐091143, LF‐3, or TMZ for drug treatments.

### 2.3 Plasmids and Cell Transfection

The full‐length sequences of EN2 and SREBP1 were cloned into the overexpression construct (pcDNA3.1). ShEN2, ShSREBP1, ShTCF4, ShTCF3, ShTEF1, ShTCF1 and control shNC were synthesised by GenePharma. Lipofectamine 3000 was utilised as the reagent for cell transfection. Briefly, cells were cultured to 60%–80% confluence and then added to the plasmid together at a ratio of 1:1 with Lipofectamine 3000.

### 2.4 Immunohistochemical (IHC) Analysis

The GBM tissue samples were fixed with 4% paraformaldehyde and then embedded with paraffin wax. After the sections were dewaxed and rehydrated, the samples were immersed in 3% H_2_O_2_ and closed with 5% serum at 37°C for 30 min. Rabbit EN2 antibody (1:2000; Abcam) and Rabbit Ki67 antibody (1:2000; Abcam) were incubated at 4°C overnight, followed by Goat anti‐Rabbit IgG (1:2000, Beyotime) as the secondary antibody, incubated at room temperature for 1 h. The signals were analysed with a light microscope.

### 2.5 Quantitative Real‐Time Polymerase Chain Reaction (qRT‐PCR)

All RNA was obtained from GBM cells by using Trizol reagent (Ambion). Next, the obtained RNA was reverse‐transcribed into cDNA using the reverse transcription system kit (Genepharma) for subsequent experiments. SYBR Green Mix (Genepharma) was performed on a fluorescence quantitative PCR apparatus to evaluate the mRNA phase pair expression level. The relative expression of interest genes was normalised to the expression of GAPDH, and the relative expression of the target gene was determined by 2^−ΔΔCt^.

### 2.6 Western Blot Assay

Total protein of the GBM cells was extracted with RIPA buffer containing protease inhibitors (1:100; Beyotime) for ice cracking for 40 min and centrifuging at 12,000 rpm for 10 min. Then the concentration was measured with the BCA kit. After that, a protein marker and an equal amount of protein sample were added into the hole of 10% Tris‐Bis gel, and electrophoresis was conducted at 110 V. Sealed with skim milk at room temperature for 2 h. Then, rabbit EN2 (1:2000; Abcam) and rabbit SREBP1 (1:2000; Abcam) were incubated at 4°C overnight. Mouse GAPDH was used as the internal control. Finally, chemiluminescence was performed, and imaging was taken.

### 2.7 CCK8 and EDU Assays

5 × 10^3^ GBM cells were implanted in each 96‐well plate, and then 10 μL CCK‐8 solution was added to each well at 0, 24, 48, 72 and 96 h and incubated in an incubator at 37°C for 2 h. After that, an enzyme labeller was used to obtain the absorbance value at a 450 nm wavelength. GBM cells were inoculated in 24‐well plates for EdU detection. After 24 h, the EDU cell proliferation test was performed according to the instructions using the EDU cell proliferation kit (Beyotime) and AlexaFluor555. In addition, the nuclei were stained with DAPI (Beyotime) at room temperature for 10 min. Finally, fluorescence microscopy was used to observe the stained cells and calculate the proportion of EDU‐positive cells.

### 2.8 Colony Formation Assay

1 × 10^3^ GBM cells were added to each 6‐well plate (NEST) and cultured in a 5% CO_2_ incubator at 37°C. After 14 days, the cell colonies were gently washed with PBS, then fixed with 4% paraformaldehyde (Solarbio) at room temperature for 20 min, and then stained with 0.5% crystal violet (Solarbio) for 30 min. Colonies of 50 cells were selected, the rate of clone formation was assessed, and images were obtained using a gel imaging system.

### 2.9 The Transwell Assay

GBM cells were first collected and resuspended in a serum‐free medium in a matrix gel mixture. Transwell chambers were inserted into a 24‐well plate containing 20% serum. After incubating in a 5% CO_2_, 37°C incubator for 24 h, the remaining cells on the upper surface were removed with cotton swabs, and then the adherent cells on the lower surface were fixed with 4% paraformaldehyde and stained with crystal purple. Finally, the number of infiltrating cells was observed by microscopy.

### 2.10 Lipid Drop Detection

First, the GBM cells on the cell slide were fixed with 4% paraformaldehyde at room temperature for 15 min, then stained with BODIPY 493/503 for 30 min. Next, the samples were stained with DAPI at room temperature for 6 min and then washed with PBS at least 3 times. Finally, the images were captured by confocal microscope, and the lipid droplet content was quantitatively and statistically analysed by ImageJ software.

### 2.11 Chromatin Immunoprecipitation‐qPCR Assay

Starting with the EZ ChIP kit according to the manufacturer's instructions. The GBM cells are cross‐linked and formed into balls, and the lytic supernatant is treated ultrasonically. The supernatant was incubated with protein G beads, 10 μg anti‐TCF4 antibody, or 10 μg rabbit IgG antibody to detect the pulled and purified DNA fragments.

### 2.12 RNA Sequencing and Transcriptome Analysis

RNA libraries were constructed from each experimental group, including three biological replicates per sample. The sequencing platform utilised for poly(A) and RNA isolation, library construction and sequencing was the MGISEQ‐2000RS. The sequence quality of all samples was assessed using FastQC (v0.11.7), and quality pruning was performed utilising the FASTX‐Toolkit (v0.0.14). Gene expression profiles were analysed based on read count data. Using SAMtools (v1.7) and HTSeq‐count (v0.9.1), expression values and transcription levels for each gene were estimated as Fragments Per Kilobase of Exon per Million Mapped Reads (FPKM). Differentially expressed genes (DEGs) were identified using DESeq2 (v1.30.1), where a significance threshold of *p* ≤ 0.05 and an absolute fold change of ≥ 1 were applied.

### 2.13 Dual‐Luciferase Assay

First, the cDNA fragment containing the EN2 promoter region was amplified by PCR and then cloned into the pGLO luciferase reporter plasmid. GBM cells with the silence of TCF4 were seeded in a 24‐well plate and cultured overnight, followed by transfection of the recombinant construct into the cells using Lipofectamine 3000. Finally, the luciferase reporter assay kit was used to detect luciferase activity.

### 2.14 IC50 Determination Assay

First, four groups were set up: the blank control group, the EN2 silence only group, the TMZ treatment only group and the group with both EN2 silence and TMZ treatment. A total of 1 × 10^3^ GBM cells were seeded in a 96‐well plate and cultured at 37°C in 5% CO_2_. The samples were collected at different time points, such as 0, 24, 48, 72 and 96 h. GBM cells were fixed with 4% paraformaldehyde at room temperature for 20 min and then stained with 0.1% crystal violet for 30 min. Finally, absorbance values (OD) were measured at 450 nm using a microplate reader, and a curve was plotted.

### 2.15 Animal Studies

All animal experiments were approved by the Animal Ethics Committee of Ningbo University and conducted in accordance with the committee's guidelines. BALB/c nude mice (4–6 weeks old; 20.0 ± 2.0 g; male) bought from Beijing Weitong Lihua Experimental Animal Technology Co. Ltd. and kept under specific pathogen‐free conditions. Transfected with shEN2 or shNC cells (2 × 10^5^ cells per mouse, 0.2mL PBS), they were injected subcutaneously into the lower back of mice. Tumour size was measured every 7 days, and tumour mass was measured after 28 days. Tumour volume size (mm^3^) was measured using a vernier calliper and calculated as: volume = length × width^2^ × 0.5. When the tumour diameter reached 5 mm, the mice were randomly divided into treatment groups with 5 mice in each group. The mice were killed at 28 days, and the tumours and their organs (heart, lung, liver, spleen, kidney) were dissected. The paraffin‐embedded sections were dewaxed and stained with H&E and IHC.

### 2.16 Statistical Analysis

We used GraphPad Prism 8 software for the statistical analysis of the experimental data during the study. Data were expressed as mean ± standard deviation. The experiments were statistically significant when *p* < 0.05. * indicates *p* < 0.05; ** indicates *p* < 0.01; *** denotes *p* < 0.001.

## 3 Results

### 3.1 The Elevated Expression of EN2 in GBM Is Associated with Poor Prognosis in Patients

**FIGURE 1:**
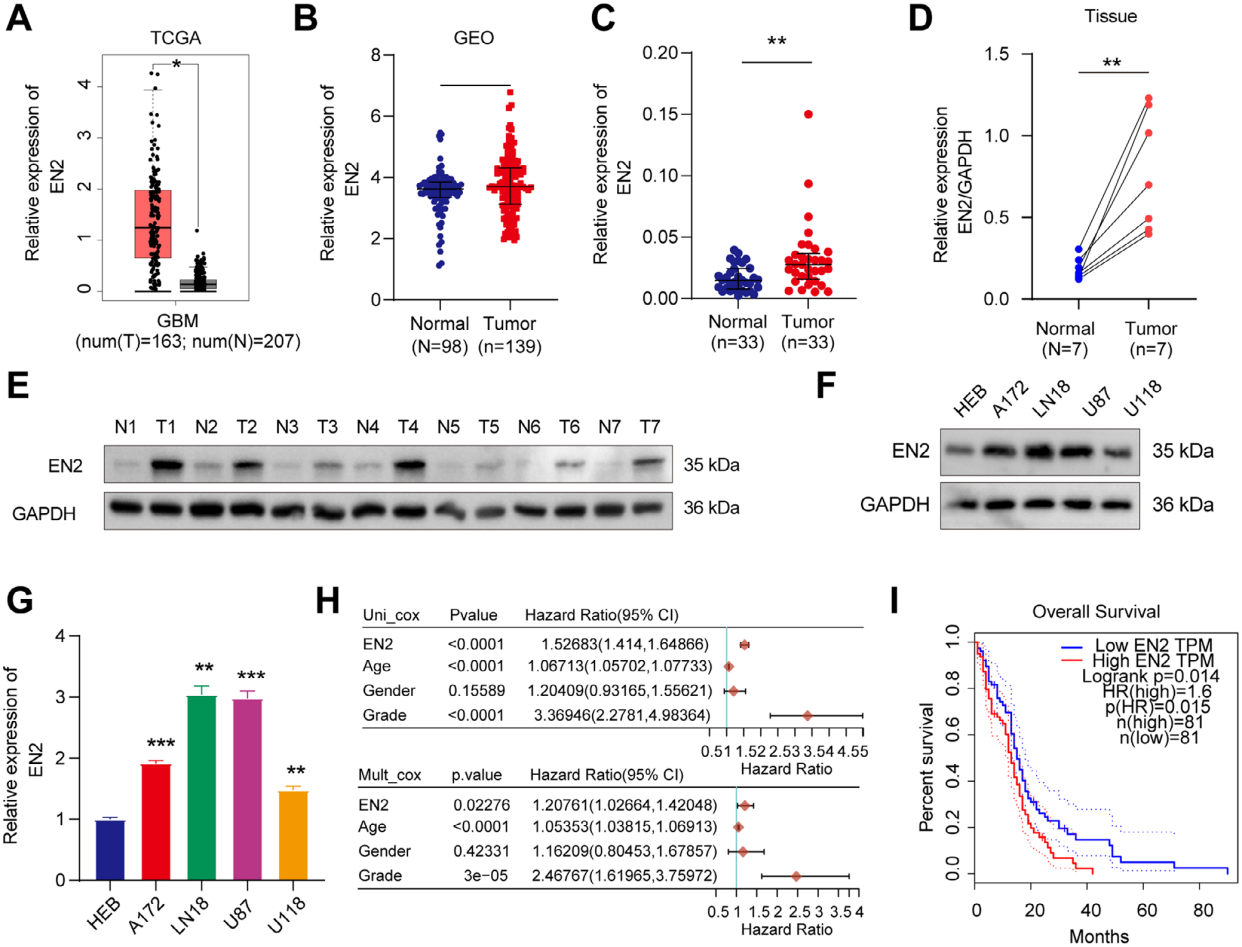
The high expression of EN2 in GBM was associated with poor prognosis in patients. (A) The TCGA database predicted the expression level of EN2 was observably increased in GBM tumour tissues. (B) The GEO database predicted the expression level of EN2 was observably increased in GBM tumour tissues. (C) qRT‐PCR detection indicated the expression level of EN2 was observably increased in GBM tumour tissues. (D, E) Western blot assay indicated the expression level of EN2 was observably increased in GBM tumour tissues. (F, G) qRT‐PCR detection indicated the expression level of EN2 was observably increased in GBM cell lines. (H) Univariate and multivariate Cox regression analysis for the *p*‐values, risk coefficients HR, and confidence intervals of the clinical features and the expression of EN2, indicating EN2 as a variable independent of other clinical factors. (I) The KM survival curve for EN2 in TCGA data, where log‐rank tested between different groups, indicated that the higher expression of EN2 correlated with poorer prognosis in patients. Data information: In all relevant panels, ns, no significant; *p < 0.05; **p < 0.01; ***p < 0.001; two‐tailed *t*‐test. Data are presented as mean ± SD and are representative of three independent experiments.

To further investigate the expression of EN2 in GBM tissues and its potential clinical significance, we conducted a systematic analysis of EN2 expression in GBM tumour tissues using both the TCGA and GEO databases. The results indicated that the expression of EN2 in GBM tumour tissues was significantly higher than that in normal tissues (Figure 1A,B). Subsequently, to validate the high expression of EN2 observed in the databases, we directly assessed EN2 expression levels in GBM tumour tissues using qRT‐PCR and Western blot. As anticipated, EN2 expression was significantly elevated in GBM tumour tissues (Figure 1C–E). Additionally, we validated EN2 expression in various GBM cell lines, including A172, LN18, U87 and U118. The qRT‐PCR and Western blot analysis revealed that EN2 expression was significantly elevated in all tested GBM cell lines (Figure 1F,G). These results further support the importance of EN2 in GBM tumours, suggesting that it may play a key role in tumorigenesis and progression. Next, we conducted univariate and multivariate Cox regression analyses on paraffin‐embedded GBM tissues to assess the correlation between EN2 expression and clinical factors. The results showed that the EN2 expression level was an independent prognostic factor for patient survival (Figure 1H). This finding indicated that EN2 was not only highly expressed in tumour tissues but might also be closely associated with clinical prognosis for patients. To further explore the specific impact of EN2 on the prognosis of GBM patients, we analysed the relationship between EN2 expression and patient survival using the TCGA database. The KM survival curve analysis revealed that higher EN2 expression in GBM patients correlated with poorer prognosis (Log‐Rank, *p* < 0.05) (Figure 1I). Overall, we found that high EN2 expression is not only a significant feature of GBM tumour tissues but also potentially serves as an important biomarker for guiding clinical decision‐making.

### 3.2 EN2 Promotes the Proliferation of GBM Cells In Vitro

**FIGURE 2:**
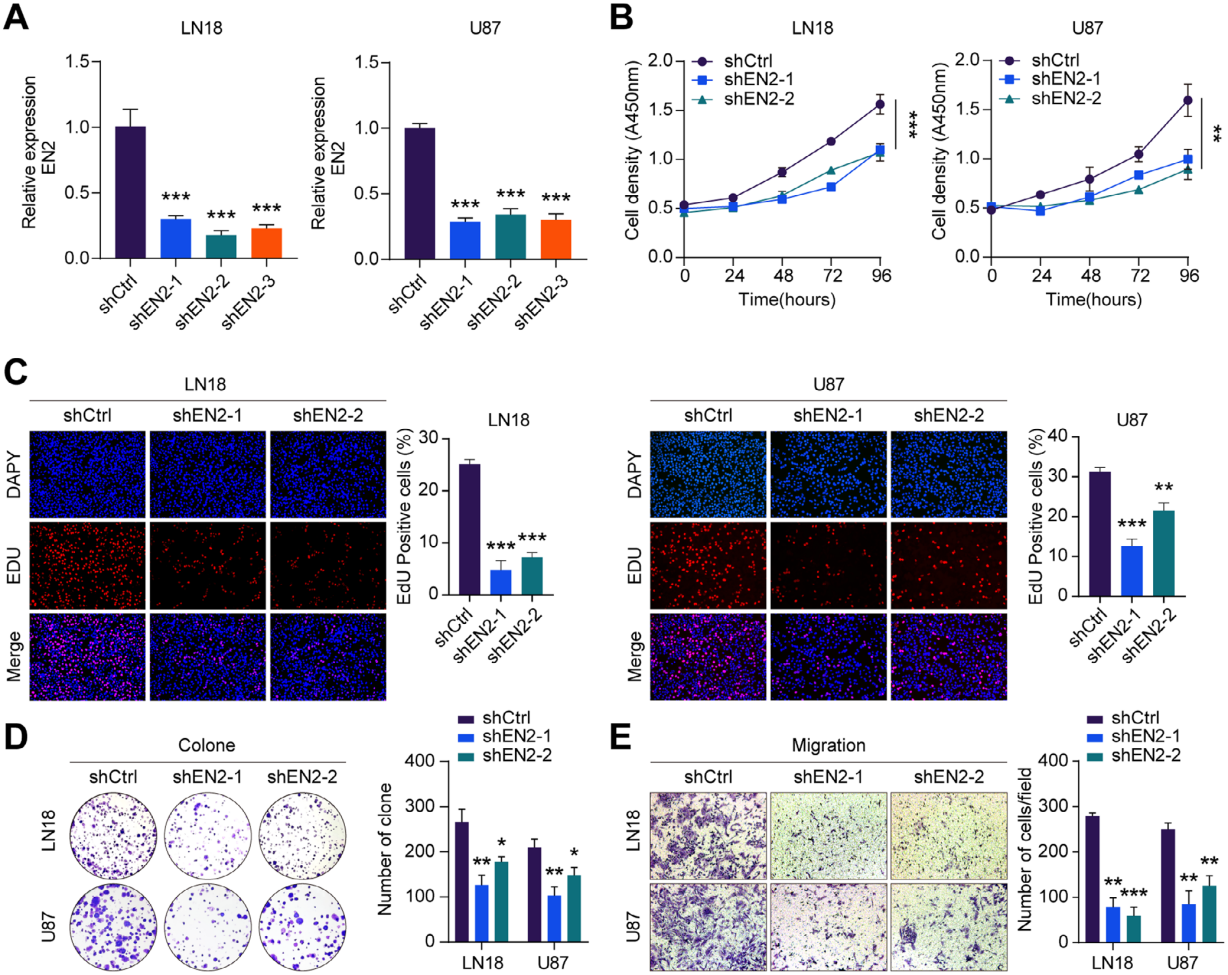
EN2 promotes the proliferation of GBM cells in vitro. (A) After silencing EN2 in GBM cells, the expression of EN2 was detected by qRT‐PCR. (B–D) CCK8, EDU and the colony formation assays revealed that knocking down EN2 inhibited the proliferation of GBM cells. (E) The Transwell assay indicated that knocking down EN2 inhibited the invasion of GBM cells. Data information: In all relevant panels, ns, no significant; *p < 0.05; **p < 0.01; ***p < 0.001; two‐tailed *t*‐test. Data are presented as mean ± SD and are representative of three independent experiments.

To further investigate the specific function and role of EN2 in GBM cells, we transfected LN18 and U87 cells with three independent shRNAs to knock down EN2. To verify the successful knockdown of EN2 in GBM cells, we assessed the expression of EN2 in these cells. qRT‐PCR showed that the expression of EN2 in LN18 and U87 cells significantly decreased after knocking down EN2 (Figure 2A). This also confirmed that we successfully inhibited the expression of EN2. Next, we conducted several cell proliferation experiments to explore the impact of EN2 on the proliferation capacity of GBM cells, including the CCK‐8 assay, EDU assay and colony formation assay. The CCK‐8 assay showed that the viability of LN18 and U87 cells decreased after knocking down EN2 (Figure 2B). Moreover, the result from the EDU assay was consistent with that of the CCK‐8 assay, indicating a reduced proliferation capacity of LN18 and U87 cells after knockdown of EN2 (Figure 2C). Notably, the colony formation assay also revealed that the density of LN18 and U87 cells in the field was significantly lower than that of the control group with knocking down EN2, suggesting a marked inhibition of their proliferative capacity (Figure 2D). All these findings consistently indicated that the proliferation levels of LN18 and U87 cells were significantly reduced after knocking down EN2 compared to the control group. Subsequently, we evaluated the impact of knocking down EN2 on the invasive capacity of GBM cells using the Transwell assay. The results showed that the migration ability and invasion numbers of LN18 and U87 cells were significantly lower than those of the control group after knocking down EN2 (Figure 2E). In summary, our research findings suggest that EN2 not only promotes the proliferation of GBM cells in vitro but also enhances their invasive ability, indicating that EN2 plays an important role in the occurrence and development of GBM.

### 3.3 EN2 Promotes the Proliferation of GBM Cells In Vivo

**FIGURE 3:**
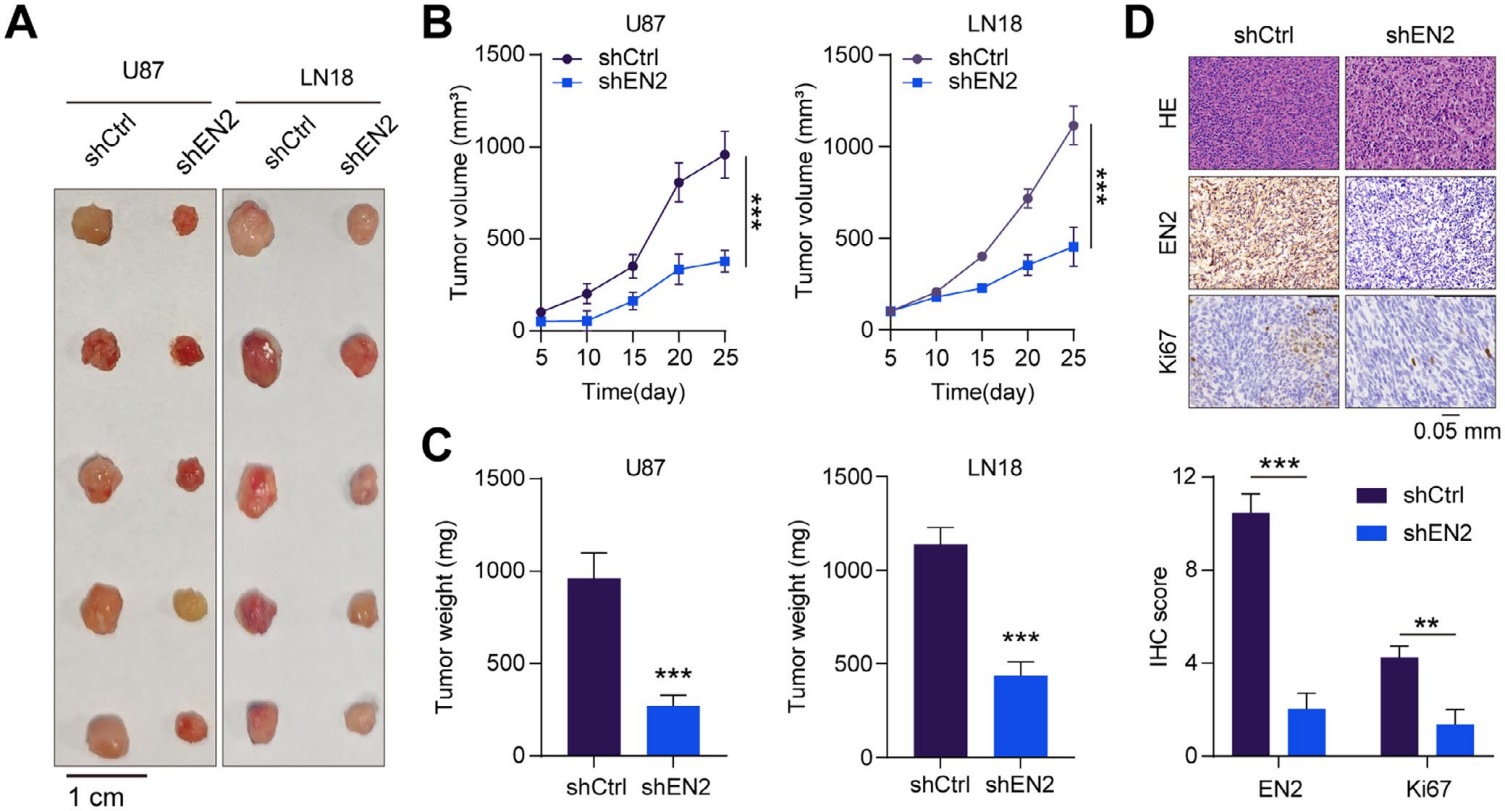
EN2 promotes the proliferation of GBM cells in vivo. (A) Subcutaneous injection of U87 and LN18 glioblastoma cells with either shNC or shEN2 in nude mice resulted in tumour formation, and the tumours were harvested after 25 days. (B) After subcutaneous injection of GBM cells, tumour volumes were measured every 5 days, and a growth curve was plotted. (C) Weighing of the tumours. (D) Immunohistochemical staining detected the expression of Ki‐67 and EN2 in the tumours. Data information: In all relevant panels, ns, no significant; *p < 0.05; **p < 0.01; ***p < 0.001; two‐tailed *t*‐test. Data are presented as mean ± SD and are representative of three independent experiments.

To further elucidate the pro‐tumorigenic role of EN2, we performed transfections of U87 and LN18 GBM cells with either shNC or shEN2. Subsequently, these cells were injected subcutaneously into the left dorsal flanks of nude mice to assess the impact of EN2 knockdown on tumorigenesis. We monitored the tumour volume in the nude mice every 5 days and measured the tumour weight after 25 days. The result indicated that the silence of EN2 observably inhibited tumour growth, leading to a marked reduction in both the tumour volume and weight in the nude mice (Figure 3A–C). Additionally, we performed further analysis of the xenograft GBM tumour tissues using HE and immunohistochemistry staining, which revealed that the expression of Ki‐67 and EN2 in the tumour tissues was observably reduced (Figure 3D). In summary, these results indicated that EN2 played a critical role in the growth of GBM cells in vivo.

### 3.4 EN2 Activates Fatty Acid Synthesis Metabolism in GBM

**FIGURE 4:**
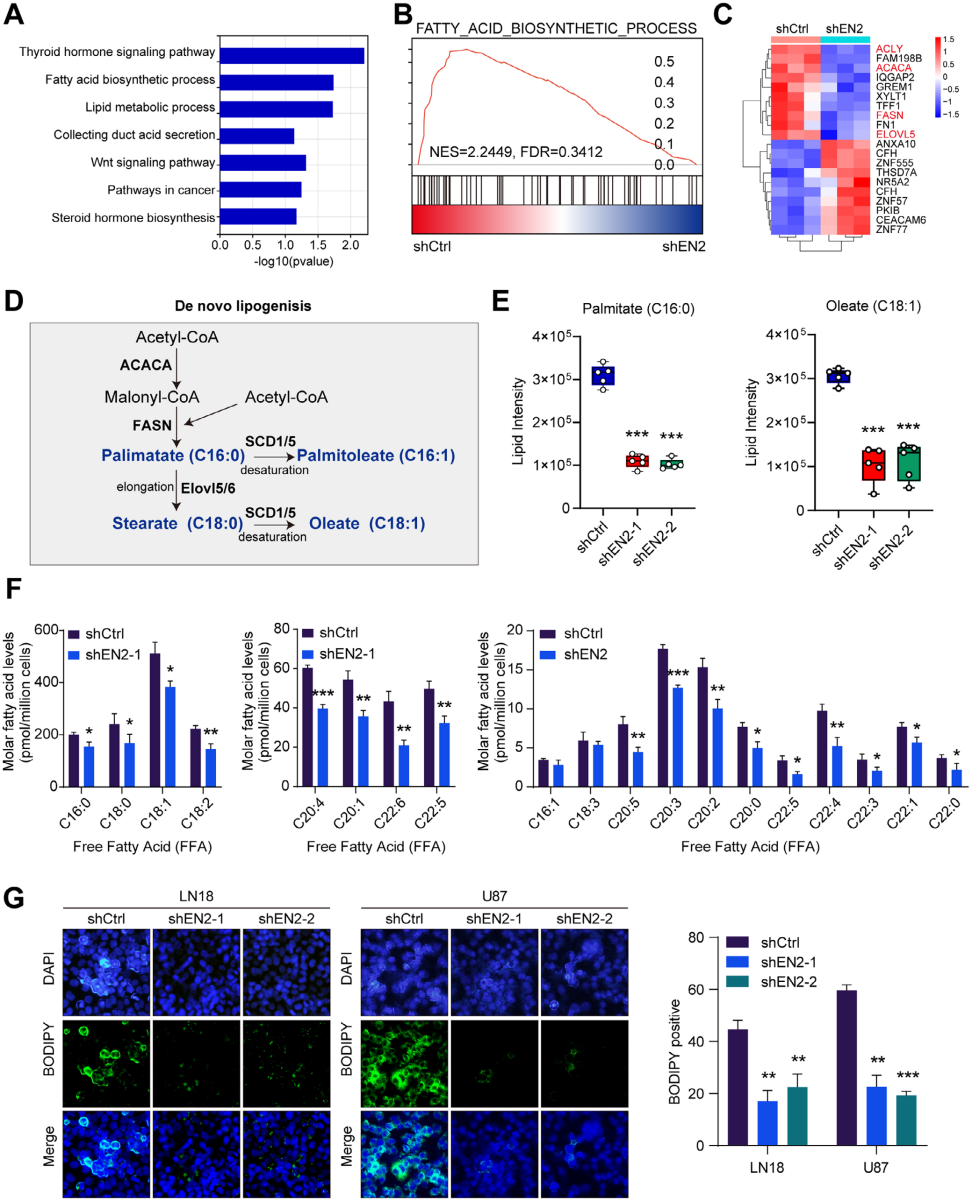
EN2 activates fatty acid synthesis metabolism in GBM cells. (A–C) Transcriptome sequencing was performed after silencing EN2, with KEGG analysis, clustering heatmap and GSEA enrichment analyses revealing an association between EN2 and fatty acid metabolic pathways. (D) Schematic diagram of the fatty acid synthesis metabolic pathway. (E) Lipid metabolomics sequencing indicated changes in palmitic acid and oleic acid ester after silencing EN2. (F) Metabolomics analysis indicated reduced levels of related products in the fatty acid synthesis pathway after silencing EN2. (G) BODIPY staining demonstrated a decrease in intracellular fatty acid content after silencing EN2. Data information: In all relevant panels, ns, no significant; *p < 0.05; **p < 0.01; ***p < 0.001; two‐tailed *t*‐test. Data are presented as mean ± SD and are representative of three independent experiments.

To further explore the molecular mechanism of EN2 on the growth of GBM cells, we transfected shEN2 and shNC into LN18 and U87 cells, followed by transcriptomic sequencing. The volcano plot indicated that there were numerous DEGs (Figure S1A). Notably, by conducting KEGG and GSEA enrichment analyses, we found that the silence of EN2 primarily affects the fatty acid synthesis metabolic pathway in GBM cells (Figure 4A,B). In addition, the clustering heatmap also indicated a decreased expression level of several key enzymes involved in the fatty acid synthesis pathway (Figure 4C). To further confirm this phenomenon, we detected the expression levels of ACLY, ACACA, FASN and ELOVL5 in GBM cells by qRT‐PCR. As expected, the mRNA expression levels of these four key enzymes involved in fatty acid synthesis were observably reduced, indicating that EN2 influenced fatty acid synthesis in cancer cells (Figure S1B). Subsequently, we also verified a positive correlation between the mRNA expression of EN2 and ACLY, ACACA, FASN and ELOVL5 using the TCGA database (Figure S1C). Acetyl‐CoA is an important substrate for fatty acid synthesis, and ACACA, FASN and ELOVL5 all play critical roles in this process (Figure 4D). Based on this, we conducted lipid metabolomics sequencing, and the results showed a significant decrease in the levels of palmitic acid and oleic acid ester, as well as a general reduction in various free fatty acids (Figure 4E,F). To validate this finding, we used BODIPY staining to assess changes in overall fatty acid level in GBM cells. The result revealed that after the silence of EN2, the fatty acid content in GBM cells observably decreased (Figure 4G). In summary, we found that EN2 played an important role in the fatty acid synthesis metabolism of GBM cells, promoting the generation of fatty acids in cancer cells.

### 3.5 EN2 Activates Fatty Acid Synthesis Metabolism by Upregulating SREBP1 to Promote the Growth of GBM Cells

**FIGURE 5:**
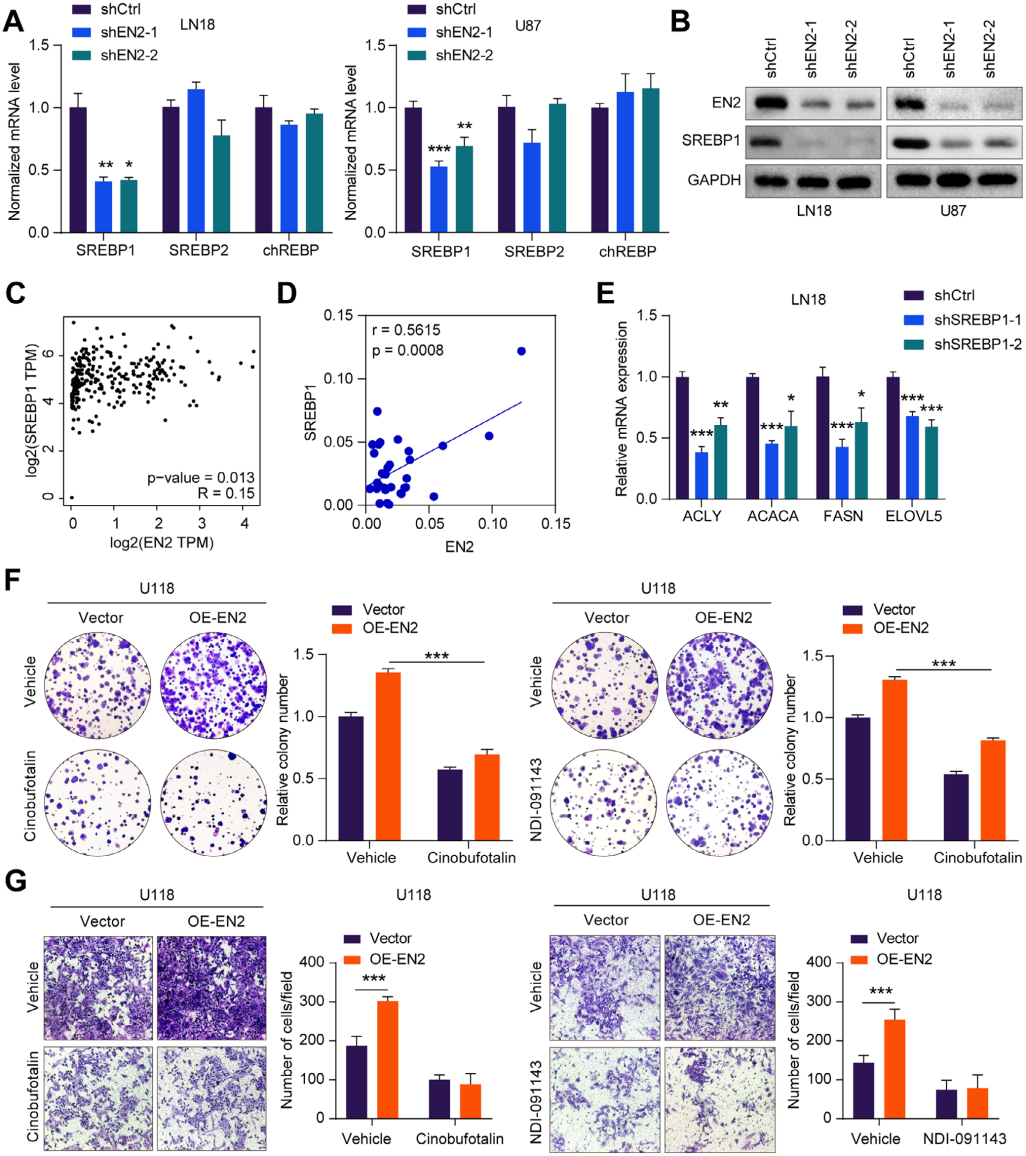
EN2 activates fatty acid synthesis metabolism by upregulating SREBP1 to promote the growth of GBM cell. (A, B) qRT‐PCR and Western blot detection revealed that knocking down EN2 downregulated the expression of SREBP1. (C, D) The analysis of the TCGA database and clinical samples showed a positive correlation between EN2 and SREBP1. (E) Western blot detection revealed that protein expression of SREBP1 decreased after knocking down EN2. (F) The colony formation assay indicated that the addition of the SREBP1 inhibitor cinobufotalin or the ACLY inhibitor NDI‐091143 could diminish the enhancement of the proliferation induced by the overexpression of EN2 in GBM cells. (G) The Transwell assay indicated that the addition of the SREBP1 inhibitor cinobufotalin or the ACLY inhibitor NDI‐091143 could diminish the enhancement of the invasive capacity induced by the overexpression of EN2 in GBM cells. Data information: In all relevant panels, ns, no significant; *p < 0.05; **p < 0.01; ***p < 0.001; two‐tailed *t*‐test. Data are presented as mean ± SD and are representative of three independent experiments.

Previous studies have indicated that SREBP is a key transcription factor regulating the expression of genes related to lipid synthesis and uptake. SREBP1 plays an important role in controlling and regulating fatty acid and cholesterol synthesis [23]. To further investigate how EN2 activated fatty acid synthesis metabolism in GBM cells to promote their proliferation, we detected the expression levels of genes involved in the control and regulation of fatty acid synthesis by qRT‐PCR. The result indicated that knocking down EN2 in GBM cells observably reduced the mRNA expression level of SREBP1, while there were no biological differences in the expression of SREBP2 and chREBP (Figure 5A). Furthermore, we evaluated the change in protein expression level of SREBP1 by Western blot. As expected, the expression of SREBP1 was observably decreased with the silence of EN2 in GBM cells (Figure 5B). Notably, we also verified a positive correlation between EN2 and SREBP1 mRNA expression in both the TCGA database and clinical samples (Figure 5C,D). Subsequently, we transfected shSREBP1 and shNC into LN18 cells and U87 cells to knock down SREBP1 in GBM cells. The result indicated a significant reduction in the expression of SREBP1 in GBM cells by qRT‐PCR (Figure S2A). To validate the important role of SREBP1 in fatty acid synthesis in cancer cells, we found that the triglyceride content observably decreased in GBM cells whose EN2 was knocked down (Figure S2B). Next, we also detected the expression of key enzymes involved in fatty acid synthesis in GBM cells whose EN2 was knocked down by qRT‐PCR. As expected, the mRNA expression levels of ACLY, ACACA, FASN and ELOVL5 were observably lower compared to the control group with the silencing of EN2 in GBM cells (Figures 5E and S2D). Overall, these results indicated that SREBP1 played a crucial role in the fatty acid synthesis metabolism of GBM cells and could promote lipid generation.

To further explore the association between EN2 and SREBP1 in the fatty acid synthesis metabolic process, we overexpressed EN2 in U118 cells and A172 cells. The colony formation assay demonstrated that the overexpression of EN2 enhanced the proliferative capacity of GBM cells (Figures 5F and S2G). The Transwell assay indicated that the overexpression of EN2 promoted the invasive capacity of GBM cells (Figures 5G and S2H). Cinobufotalin, as an inhibitor of SREBP1, and NDI‐091143, as an inhibitor of ACLY, both suppressed the fatty acid synthesis metabolic process. Therefore, we treated GBM cells whose EN2 was overexpressed with cinobufotalin and NDI‐091143. To assess their effects on the proliferation and invasion capabilities of GBM cells, we validated the results using the colony formation assay and the Transwell assay. The results indicated that treatment with the SREBP1 inhibitor cinobufotalin or the ACLY inhibitor NDI‐091143 effectively diminished the enhancement of the proliferation and invasion capabilities induced by the overexpression of EN2 in GBM cells (Figures 5F,G and S2G,H). In conclusion, our study revealed that EN2 can upregulate the expression of SREBP1 to activate fatty acid synthesis metabolism, thereby promoting the growth of GBM cells.

### 3.6 Wnt Signalling Activates EN2 Expression through TCF4 to Promote GBM Progression

**FIGURE 6:**
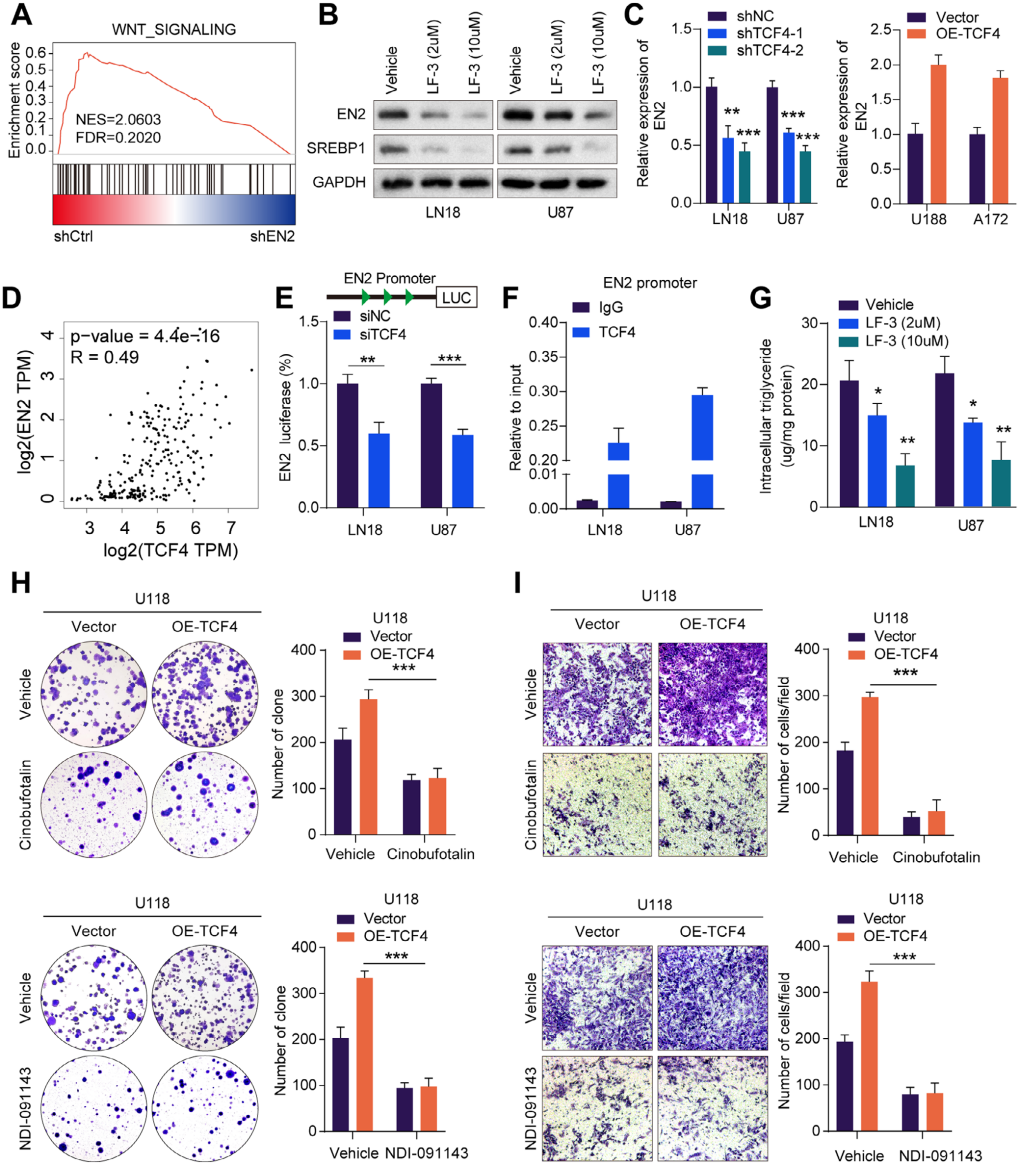
Wnt signalling activates EN2 expression through TCF4 to promote GBM progression. (A) The GSEA enrichment analysis revealed that EN2 was associated with the WNT pathway. (B) The expression levels of EN2 and SREBP1 proteins were reduced after treating cells with the Wnt inhibitor LF‐3 by Western blot. (C) Knocking down TCF4 significantly decreased the expression of EN2 by qRT‐PCR. (D) Analysis of the TCGA database revealed a positive correlation between TCF4 and EN2. (E) Dual‐luciferase assays showed a significant decrease in EN2 fluorescence intensity following the silencing of TCF4. (F) TCF4 protein was enriched on the promoter region of EN2 by ChIP‐qPCR. (G) The intracellular triglycerides were reduced after treating cells with the Wnt inhibitor LF‐3 by Western blot. (H) The colony formation assay indicated that adding the SREBP1 inhibitor cinobufotalin or the ACLY inhibitor NDI‐091143 could block the enhancement of cell proliferation caused by the overexpression of TCF4. (I) The Transwell assay showed that adding the SREBP1 inhibitor cinobufotalin or the ACLY inhibitor NDI‐091143 could block the enhancement of cell proliferation and invasion caused by the overexpression of TCF4. Data information: In all relevant panels, ns, no significant; *p < 0.05; **p < 0.01; ***p < 0.001; two‐tailed *t*‐test. Data are presented as mean ± SD and are representative of three independent experiments.

Through further analysis of the transcriptome sequencing results, we found that EN2 was associated with the Wnt signalling pathway through GSEA enrichment analysis (Figure 6A). To further investigate the relationship between the Wnt signalling pathway and EN2, we treated LN18 cells and U87 cells with the Wnt inhibitor LF‐3. It revealed that the protein expression levels of EN2 and SREBP1 observably decreased in GBM cells after LF‐3 treatment by Western blot (Figure 6B). Additionally, we examined the expression of key fatty acid synthesis enzymes in GBM cells treated with LF‐3. The results indicated that the expression levels of ACLY, ACACA, FASN and ELOVL5 were observably reduced by qRT‐PCR (Figure S3A). Previous studies have indicated that during the activation of Wnt/nt/ (Figure S3A). Previous studies translocate from the cytoplasm to the nucleus and bind with the TCF/LEF family, thereby regulating the transcription of Wnt target genes [24]. Among the TCF/LEF family, TCF4 is the most important transcription factor [19]. Next, we successfully knocked down TCF4, TCF3, TEF1, TCF1 and the negative control (shNC) in GBM cells by transfection. We found that after knocking down TCF4 in GBM cells, the expression level of EN2 observably decreased by qRT‐PCR; however, the expression level of EN2 showed no biological differences after knocking down TCF3, TEF1, or TCF1 (Figures 6C and S3B). Furthermore, we validated this in U118 and A172 cells overexpressing TCF4. As expected, the expression level of EN2 was observably upregulated after the overexpression of TCF4 (Figures 6C and S3C). Additionally, we verified a positive correlation between TCF4 and EN2 expression in the TCGA database (Figure 6D).

To further validate the relationship between TCF4 and EN2, we successfully constructed a dual‐luciferase reporter vector for the promoter of EN2. The results indicated that the fluorescence intensity in GBM cells with the silence of TCF4 was observably lower than the control group, indicating a notable decrease in the expression level of EN2 (Figure 6E). Moreover, we found that TCF4 could bind to the EN2 promoter through ChIP‐qPCR (Figure 6F). Therefore, TCF4 played an important role in the function of EN2 within the Wnt signalling pathway.

Our study has confirmed that EN2 can upregulate the expression of SREBP1, subsequently activating fatty acid synthesis metabolism. What is the relationship between TCF4 and fatty acid synthesis metabolism? We found that disrupting the interaction between β‐catenin and TCF4 by LF‐3 significantly reduced the triglyceride content in GBM cells (Figure 6G). Furthermore, we validated a positive correlation between TCF4 and the expression of ACACA, ELOVL5, FASN, SREBP1 and ACLY in the TCGA database (Figure S3D). Thus, TCF4 regulated the fatty acid synthesis metabolic pathway in GBM cells. Finally, we treated U118 cells overexpressing TCF4 with the SREBP1 inhibitor cinobufotalin or the ACLY inhibitor NDI‐091143. The colony formation assay showed that this treatment effectively blocked the increased proliferation of U118 cells due to the overexpression of TCF4 (Figures 6H and S3E). The Transwell assay demonstrated that the treatment effectively inhibited the enhanced invasiveness of U118 cells due to the overexpression of TCF4 (Figures 6I and S3F). Overall, our study revealed that the Wnt signalling pathway primarily activated the expression of EN2 through TCF4 to promote fatty acid synthesis metabolism, thereby facilitating GBM progression.

### 3.7 Knocking Down EN2 Reduces the Drug Resistance of GBM Cells

**FIGURE 7:**
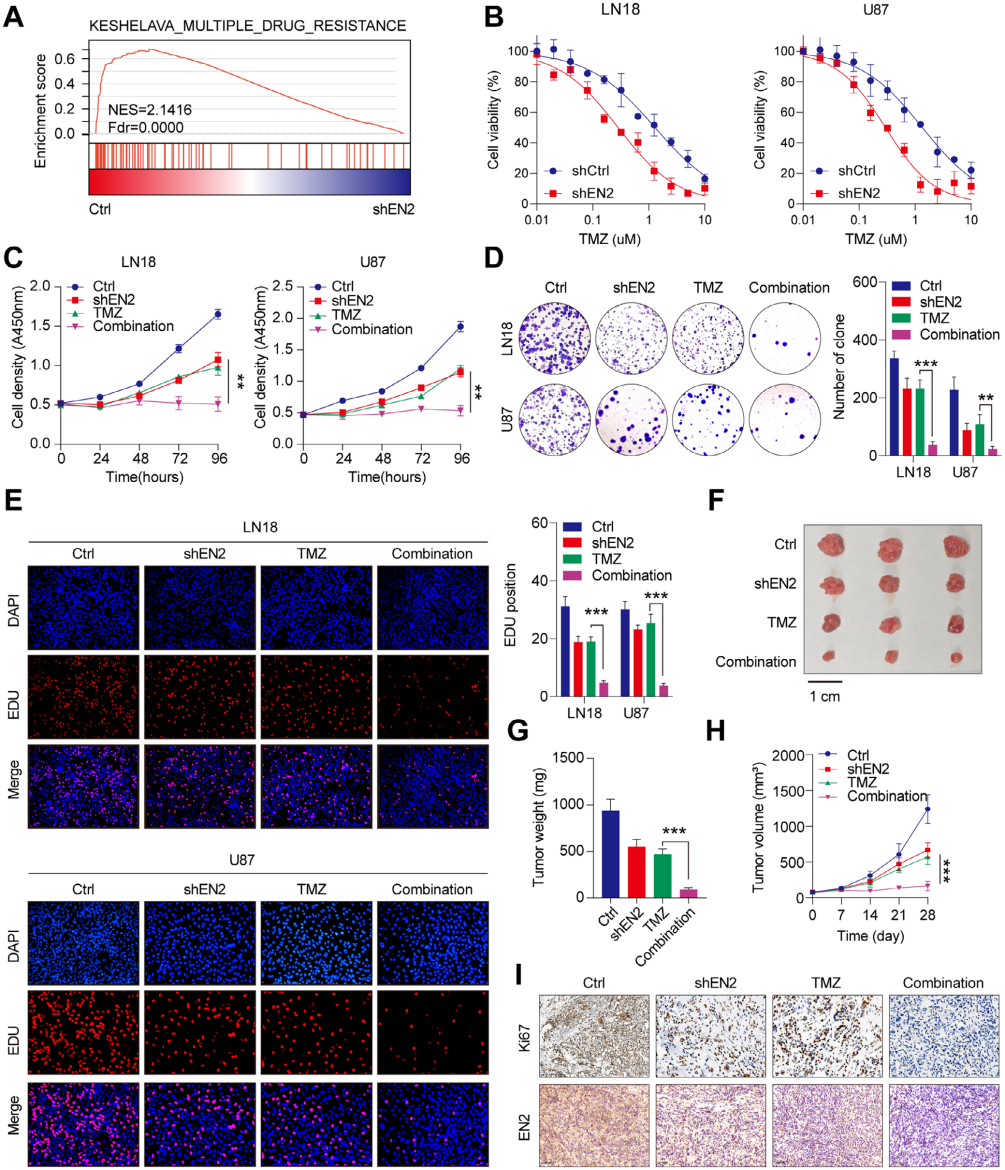
Knocking down EN2 reduces the drug resistance of GBM cells. (A) After the silence of EN2, transcriptomic sequencing and GSEA enrichment analysis revealed that EN2 was associated with multidrug resistance. (B) IC50 experiments indicated a reduction in TMZ resistance after the silencing of EN2. (C–E) CCK‐8, the colony formation and EDU assays revealed a dramatic decrease in cell proliferation ability after the silencing of EN2 and TMZ treatment. (F) After subcutaneous tumour implantation in nude mice, drug treatment was performed. (G) After 28 days, the mice were euthanised, and the tumours were harvested for weighing. (H) The sizes of tumours were measured every 7 days and plotted. (I) Immunohistochemical analysis of Ki‐67 and EN2 revealed that drug treatment significantly inhibited protein expression after the silence of EN2. Data information: In all relevant panels, ns, no significant; *p < 0.05; **p < 0.01; ***p < 0.001; two‐tailed t‐test. Data are presented as mean ± SD and are representative of three independent experiments.

Through further analysis of the transcriptomic sequencing results, we found that EN2 was associated with multidrug resistance through GSEA enrichment analysis (Figure 7A). Temozolomide (TMZ) is an orally administered alkylating agent and is considered a first‐line chemotherapy drug for the treatment of GBM [25]. To further investigate the relationship between EN2 and drug resistance, IC50 experiments revealed that GBM cells with the silence of EN2 exhibited reduced resistance to TMZ when treated with various concentrations of TMZ (Figure 7B). Subsequently, we treated GBM cells with the drug TMZ. The CCK‐8 assay, colony formation assay and EDU assay indicated a dramatic decrease in the proliferation ability of GBM cells with the silencing of EN2 (Figure 7C–E). Therefore, knocking down EN2 effectively reduced the drug resistance of GBM cells in vitro. To further explore the drug resistance of GBM cells with the silence of EN2 in vivo, we subcutaneously implanted GBM cell‐shNC and GBM cell‐shEN2 into nude mice and administered TMZ treatment, regularly monitoring the size of the tumour. Notably, we measured the size of the tumour every 7 days and plotted growth curves. After 28 days, we euthanised the nude mice and harvested the tumours for weighing. The results indicated that compared to the control group, tumours in mice with only the silence of EN2 or only TMZ treatment grew slightly more slowly, while tumours in mice with both the silence of EN2 and TMZ treatment exhibited significant suppression of growth (Figure 7F–H). Overall, knocking down EN2 effectively reduced the drug resistance of GBM cells in vivo. Furthermore, we performed IHC staining on the xenograft GBM tumour tissues for further analysis, which revealed that TMZ treatment observably suppressed the expression of Ki‐67 after the silence of EN2 (Figure 7I), indicating a significant reduction in tumour growth. Thus, our study found that knocking down EN2 Reduced the drug resistance of GBM cells to TMZ.

## 4 Discussion

GBM is the most common and aggressive primary brain tumour in adults [26]. Despite the various treatment options currently available, the median survival time for patients with GBM is generally only 12–15 months, and the prognosis is relatively poor [2, 27]. Lipid metabolism reprogramming plays a crucial role in tumour initiation and progression, particularly the de novo biosynthesis of fatty acids, which can serve as a hallmark of cancer development [4, 28, 29]. Recent studies suggest that investigating fatty acid metabolism in GBM cells may reveal new drug targets and strategies for combination therapy, representing a new trend in research with the hope of improving therapeutic outcomes [30].

Several studies have confirmed that EN2 plays an important role in various malignant tumours, including colorectal cancer [9], prostate cancer [10], bladder cancer [31] and gliomas [32]. EN2, a gene containing a homeobox, is an important transcription factor that plays a key role in embryonic development and tumorigenesis [7]. In this study, we found that EN2 plays a significant role in the Wnt signalling pathway, which in turn affects the lipid metabolism reprogramming of GBM, providing new insights into overcoming resistance in GBM treatment. Through clinical data analysis and in vitro and in vivo experiments, we found that EN2 observably promotes the occurrence and progression of GBM. Notably, transcriptomic sequencing results from GBM cells with the silence of EN2 showed a significant activation of the fatty acid synthesis metabolic pathway in cancer cells. Additionally, through the analysis of combined metabolomics sequencing results, we found that the fatty acid content and the levels of intermediates in fatty acid synthesis metabolism were observably reduced in GBM cells. Among various metabolic processes, especially fatty acid metabolism, play a crucial role in the proliferation and migration of cancer cells [33]. Importantly, our animal experiments indicated that knocking down EN2 in tumour tissues could observably enhance tumour resistance. In summary, EN2 plays a significant role in GBM, primarily by activating fatty acid synthesis metabolism in GBM cells to promote their proliferation.

SREBP1 is the main transcription factor for fatty acid synthesis metabolism, primarily involved in regulating the uptake and synthesis of cholesterol and fatty acids, playing a core role in tumour metabolism [23, 34, 35]. Furthermore, enhanced fatty acid metabolism is observably associated with chemotherapy drug resistance. Related studies suggest that SREBP1 is a potential therapeutic target for overcoming cisplatin resistance in NSCLC [36]. Consistent with this, we found that SREBP1 plays an important role in the fatty acid synthesis metabolism of GBM cells. First, we verified that EN2 regulates the expression of SREBP1 through clinical data analysis, Western blotting and qRT‐PCR. Next, after treating GBM cells whose EN2 was overexpressed with cinobufotalin or NDI‐091143, we found that these treatments effectively suppressed the enhanced proliferation and invasion capacity of GBM cells induced by EN2 overexpression. Finally, knocking down SREBP1 resulted in abnormal fatty acid synthesis metabolism in GBM cells. Our studies indicate that EN2 can regulate fatty acid metabolism through SREBP1, which may be the direct mechanism activating fatty acid synthesis metabolism in GBM cells.

The Wnt signalling pathway can be divided into classical and non‐classical pathways, with the classical pathway regulating cell survival, proliferation, differentiation and migration, while the non‐classical pathway mainly affects cell polarity and migration [12, 37]. Previous studies have shown that Wnt signalling is aberrantly activated in various cancers, influencing tumour initiation and progression [38, 39]. Notably, we validated that Wnt signalling regulates the expression of EN2 through GSEA enrichment analysis and Western blotting. During the Wnt/β‐catenin signalling pathway, β‐catenin regulates the transcription of Wnt target genes by interacting with the TCF/LEF family [24]. Among the TCF/LEF family, TCF4 is the most important transcription factor [19]. Importantly, we discovered that TCF4 expression is positively correlated with EN2 expression through analysis of the TCGA database and qRT‐PCR. Therefore, we further validated the binding of TCF4 to the EN2 promoter through dual‐luciferase assays and ChIP‐qPCR. Additionally, after treating GBM cells whose TCF4 was overexpressed with cinobufotalin or NDI‐091143, we found consistent results with those previously observed in GBM cells whose EN2 was overexpressed. Overall, our research indicates that the Wnt signalling pathway activates the expression of EN2 through TCF4, influencing the proliferation of GBM cells.

In summary, our study finds that EN2 promotes GBM progression by upregulating the expression of SREBP1, which in turn activates fatty acid synthesis metabolism. This process is regulated by Wnt signalling, specifically through TCF4‐mediated activation of the expression of EN2, thereby affecting the lipid metabolism reprogramming in GBM. Therefore, our results highlight the potential of EN2 as an important therapeutic target for regulating tumour metabolism, providing new strategies to overcome drug resistance and develop combination therapies.

## Author Contributions

**Junjun Zhang:** formal analysis (lead), investigation (lead), methodology (lead), writing – original draft (lead). **Shengjun Zhou:** formal analysis (supporting), investigation (supporting). **Zifeng Dai:** formal analysis (supporting), investigation (supporting). **Fanyong Gong:** formal analysis (supporting), investigation (supporting). **Jianfei Zhang:** formal analysis (equal), funding acquisition (lead), investigation (equal), methodology (equal), writing – review and editing (lead).

## Ethics Statement

All animal experiments were approved by the Animal Ethics Committee of Ningbo University and conducted in accordance with the committee's guidelines.

## Conflicts of Interest

The authors declare no conflicts of interest.

## Supporting information


**FIGURE S1.** EN2 activates fatty acid synthesis metabolism in GBM. (A) After knocking down EN2 in cells, transcriptome sequencing was performed, and a volcano plot of differential genes was constructed. (B) qRT‐PCR detection revealed that the expression of key enzymes in fatty acid synthesis decreased after knocking down EN2. (C) TCGA expression correlation analysis showed a positive correlation between EN2 and ACLY, ACACA, FASN, ELOVL5.
**FIGURE S2.** EN2 activates fatty acid synthesis metabolism by upregulating SREBP1 to promote the growth of GBM cell. (A) Expression levels of SREBP1 were assessed by qRT‐PCR following its knockdown. (B) Cellular triglyceride content was determined using a commercial assay kit, revealing a decrease upon SREBP1 knockdown. (C) The CCK8 assay demonstrated a reduction in cell proliferation following SREBP1 knockdown. (D) qRT‐PCR analysis showed downregulation of key fatty acid synthesis enzymes after SREBP1 knockdown. (E) In vivo tumour growth was monitored, with results indicating a slowdown following SREBP1 knockdown. (F) Efficacy of EN2 overexpression was confirmed by PCR and Western blot analysis. (G–H) Clonogenic and Transwell assays revealed that the addition of SREBP1 inhibitor cinobufotalin or ACLY inhibitor NDI‐091143 abrogated the enhanced cell proliferation and invasion induced by EN2 overexpression.
**FIGURE S3.** Wnt signalling activates EN2 expression through TCF4 to promote GBM progression. (A) qRT‐PCR analysis revealed a decrease in the expression of key enzymes involved in fatty acid synthesis following treatment with the Wnt inhibitor LF‐3. (B) qRT‐PCR detection showed no significant change in EN2 RNA expression after knockdown of TCF3, TEF1, or TCF1. (C) Verification of TCF4 overexpression. (D) TCGA correlation analysis identified a positive correlation between TCF4 and ACACA, ELOVL5, FASN, SREBP1 and ACLY. (E–F) Clonogenic and Transwell assays demonstrated that the addition of SREBP1 inhibitor cinobufotalin or ACLY inhibitor NDI‐091143 could block the promotional effects of TCF4 overexpression on cell proliferation and invasion.

## Data Availability

Additional data supporting the conclusions of this study can be accessed from the corresponding author upon a reasonable request.
