# Supplementary material for: Wnt Signalling‐Activated EN2 Promotes the Progression of Glioblastoma by Upregulating Fatty Acid Synthesis Metabolism
Source: J Cell Mol Med. 2025 Sep 1;29(17):e70726. doi: 10.1111/jcmm.70726 (PMC12401138; doi:10.1111/jcmm.70726)
Supplement: Supplementary file 1 — FIGURE S1. EN2 activates fatty acid synthesis metabolism in GBM. (A) After knocking down EN2 in cells, transcriptome sequencing was performed, and a volcano plot of differential genes was constructed. (B) qRT‐PCR detection revealed that the expression of key enzymes in fatty acid synthesis decreased after knocking down EN2. (C) TCGA expression correlation analysis showed a positive correlation between EN2 and ACLY, ACACA, FASN, ELOVL5. FIGURE S2. EN2 activates fatty acid synthesis metabolism by upregulating SREBP1 to promote the growth of GBM cell. (A) Expression levels of SREBP1 were assessed by qRT‐PCR following its knockdown. (B) Cellular triglyceride content was determined using a commercial assay kit, revealing a decrease upon SREBP1 knockdown. (C) The CCK8 assay demonstrated a reduction in cell proliferation following SREBP1 knockdown. (D) qRT‐PCR analysis showed downregulation of key fatty acid synthesis enzymes after SREBP1 knockdown. (E) In vivo tumour growth was monitored, with results indicating a slowdown following SREBP1 knockdown. (F) Efficacy of EN2 overexpression was confirmed by PCR and Western blot analysis. (G–H) Clonogenic and Transwell assays revealed that the addition of SREBP1 inhibitor cinobufotalin or ACLY inhibitor NDI‐091143 abrogated the enhanced cell proliferation and invasion induced by EN2 overexpression. FIGURE S3. Wnt signalling activates EN2 expression through TCF4 to promote GBM progression. (A) qRT‐PCR analysis revealed a decrease in the expression of key enzymes involved in fatty acid synthesis following treatment with the Wnt inhibitor LF‐3. (B) qRT‐PCR detection showed no significant change in EN2 RNA expression after knockdown of TCF3, TEF1, or TCF1. (C) Verification of TCF4 overexpression. (D) TCGA correlation analysis identified a positive correlation between TCF4 and ACACA, ELOVL5, FASN, SREBP1 and ACLY. (E–F) Clonogenic and Transwell assays demonstrated that the addition of SREBP1 inhibitor cinobufotalin or ACLY inhibitor [file JCMM-29-e70726-s001.docx]

**Wnt signaling-activated EN2 promotes the progression of glioblastoma by upregulating fatty acid synthesis metabolism**

Supplemental figures


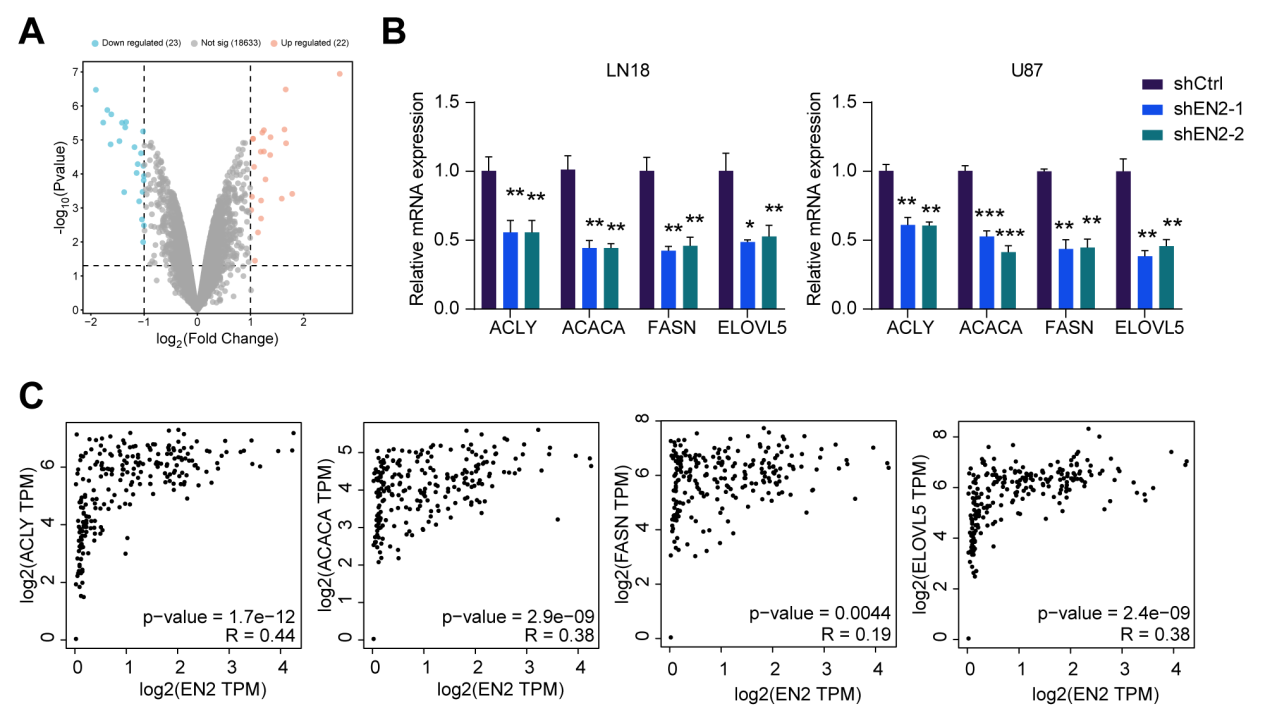


Figure S1 **EN2 activates fatty acid synthesis metabolism in GBM**

(A) After knocking down EN2 in cells, transcriptome sequencing was performed, and a volcano plot of differential genes was constructed. (B) qRT-PCR detection revealed that the expression of key enzymes in fatty acid synthesis decreased after knocking down EN2. (C) TCGA expression correlation analysis showed a positive correlation between EN2 and ACLY, ACACA, FASN, ELOVL5.


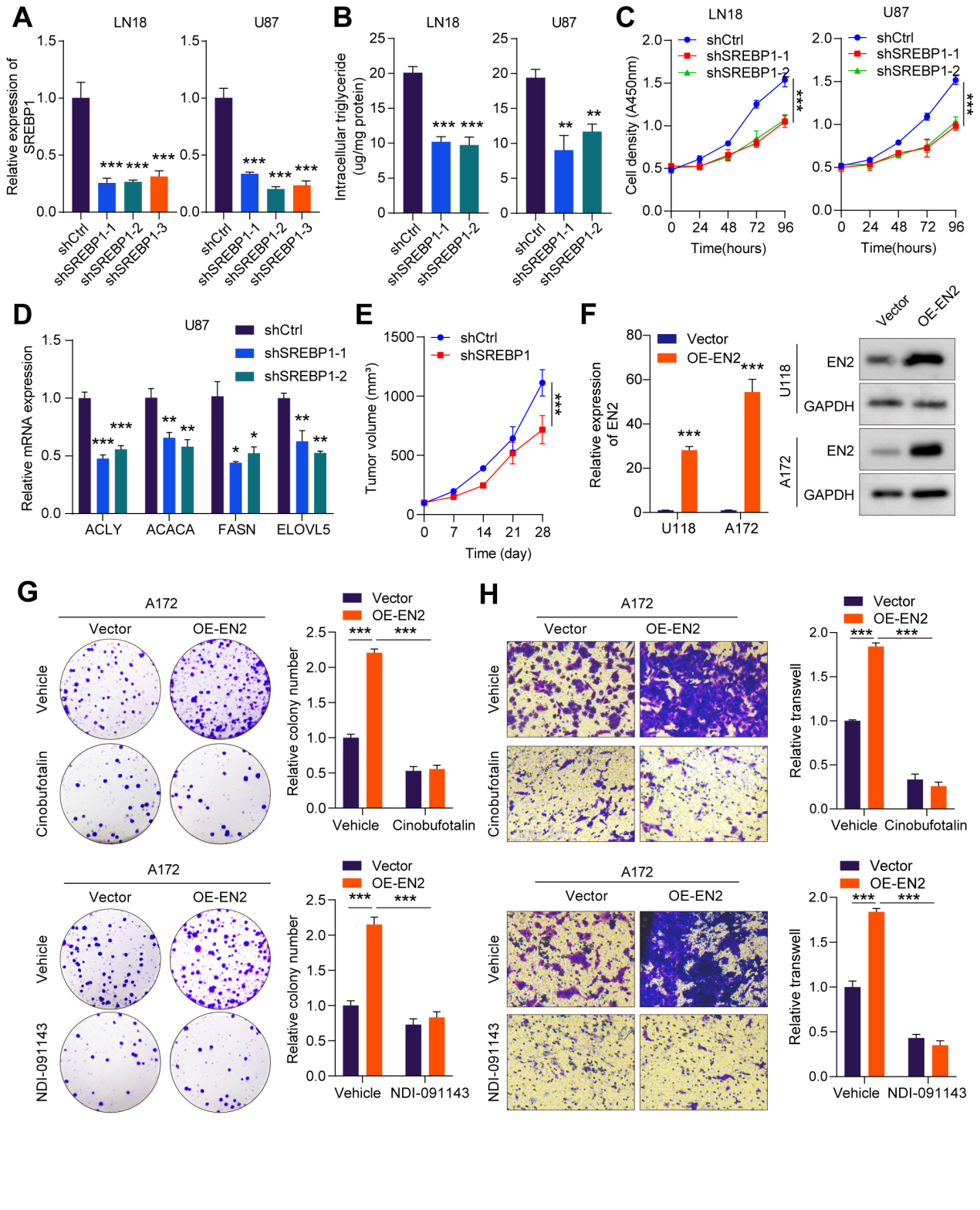


**Figure S2** **EN2 activates fatty acid synthesis metabolism by upregulating SREBP1 to promote the growth of GBM cell.**

(A) Expression levels of SREBP1 were assessed by qRT-PCR following its knockdown. (B) Cellular triglyceride content was determined using a commercial assay kit, revealing a decrease upon SREBP1 knockdown. (C) The CCK8 assay demonstrated a reduction in cell proliferation following SREBP1 knockdown. (D) qRT-PCR analysis showed downregulation of key fatty acid synthesis enzymes after SREBP1 knockdown. (E) In vivo tumor growth was monitored, with results indicating a slowdown following SREBP1 knockdown. (F) Efficacy of EN2 overexpression was confirmed by PCR and Western blot analysis. (G-H) Clonogenic and Transwell assays revealed that the addition of SREBP1 inhibitor Cinobufotalin or ACLY inhibitor NDI-091143 abrogated the enhanced cell proliferation and invasion induced by EN2 overexpression.


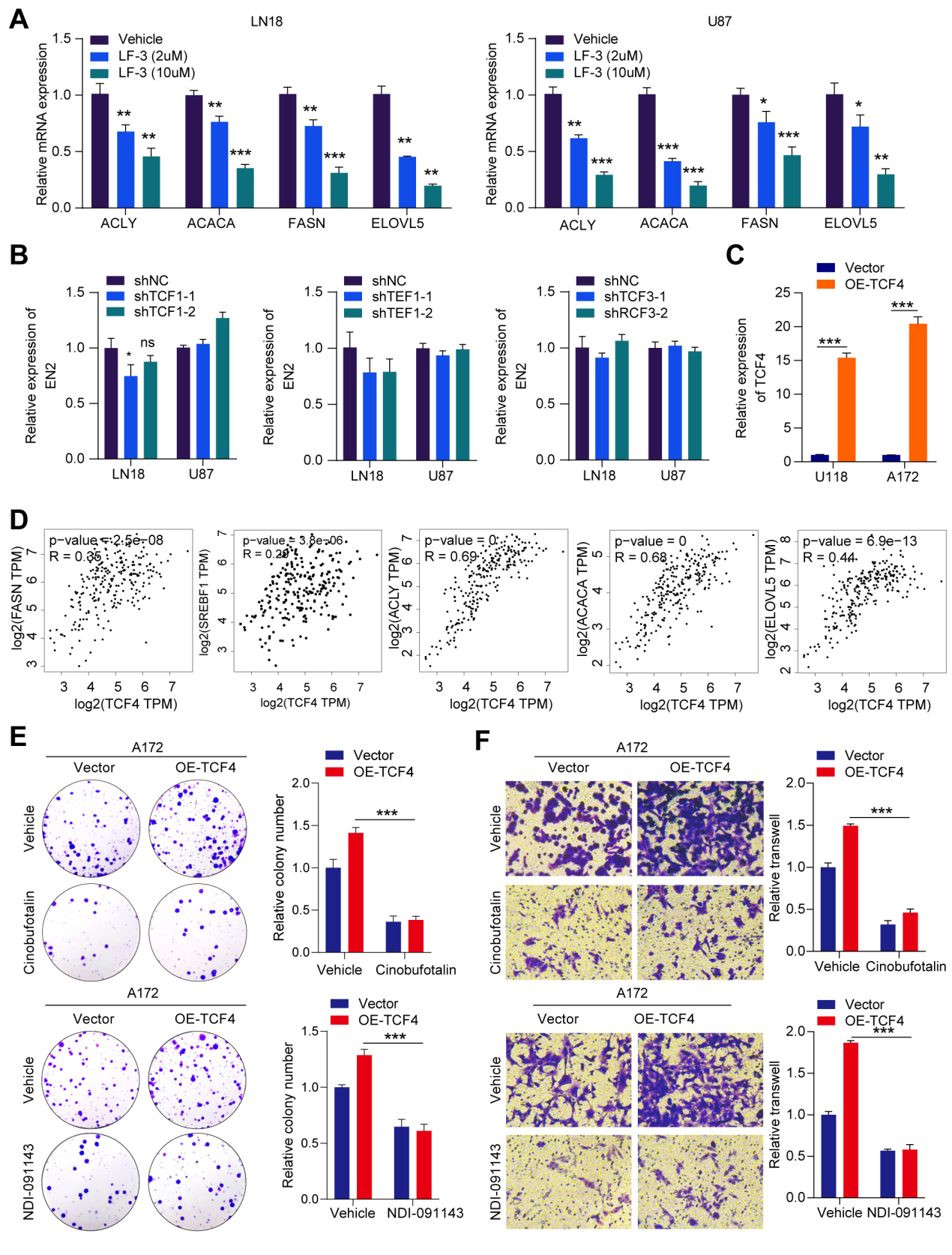


**Figure S3 Wnt signaling activates EN2 expression through TCF4 to promote GBM progression.**

(A) qRT-PCR analysis revealed a decrease in the expression of key enzymes involved in fatty acid synthesis following treatment with the Wnt inhibitor LF-3. (B) qRT-PCR detection showed no significant change in EN2 RNA expression after knockdown of TCF3, TEF1, or TCF1. (C) Verification of TCF4 overexpression. (D) TCGA correlation analysis identified a positive correlation between TCF4 and ACACA, ELOVL5, FASN, SREBP1, and ACLY. (E-F) Clonogenic and Transwell assays demonstrated that the addition of SREBP1 inhibitor Cinobufotalin or ACLY inhibitor NDI-091143 could block the promotional effects of TCF4 overexpression on cell proliferation and invasion.
